# *LMF1* frameshift deletion in Franches-Montagnes horses with hypertriglyceridemia-induced pancreatitis

**DOI:** 10.1038/s41598-025-13954-9

**Published:** 2025-08-06

**Authors:** Michaela Drögemüller, Nathalie Fouché, Michelle Wyler, Corinne Gurtner, Seraina L. Meister, Markus Neuditschko, Vidhya Jagannathan, Vinzenz Gerber, Tosso Leeb

**Affiliations:** 1 Institute of Genetics, Vetsuisse Faculty, University of Bern, 3001 Bern, Switzerland; 2 Swiss Institute of Equine Medicine, Department of Clinical Veterinary Medicine, Vetsuisse Faculty, University of Bern, 3001 Bern, Switzerland; 3 Institute of Animal Pathology, Vetsuisse Faculty, University of Bern, 3001 Bern, Switzerland; 4 Animal GenoPhenomics, Agroscope, Route de la Tioleyre 4, 1725 Posieux, Switzerland

**Keywords:** *Equus Caballus*, Metabolism, Lipid, Combined lipase deficiency, Precision medicine, Breeding, Inbreeding, Animal model, Agricultural genetics, Animal breeding, Inbreeding, Metabolic disorders

## Abstract

Hypertriglyceridemia (HTG) may be inherited and caused by variants in genes encoding enzymes of lipid metabolism. This study was prompted by the observation of eight Franches-Montagnes (FM) foals showing elevated plasma triglyceride levels and episodes of fatal acute pancreatitis. We termed this phenotype hypertriglyceridemia-induced pancreatitis (HIP). The affected foals were distantly related and inbred to a prominent stallion suggesting autosomal recessive inheritance. Whole genome sequencing of an affected foal identified a homozygous loss of function variant in *LMF1* encoding lipase maturation factor 1. The variant, XM_023616679.1:c.369_373delinsTCT, leads to an early frameshift and is predicted to alter or truncate 78% of the *LMF1* coding sequence. We genotyped the variant in a cohort of 2122 FM horses and identified 11 homozygous mutant animals including all eight foals that had initially been identified based on their clinical presentation. The three additional homozygous mutant animals had a comparable phenotype and were inbred to the same stallion. We concluded that all 11 had been affected by the same disease. Thus, we found perfect genotype-phenotype association in the tested cohort. The carrier frequency in the 2111 unaffected FM horses was 15.0%. Our findings enable genetic testing to prevent the unintentional breeding of further HIP-affected foals.

**Supplementary Information:**

The online version contains supplementary material available at 10.1038/s41598-025-13954-9.

## Introduction

Hypertriglyceridemia (HTG) comprises a heterogenous group of metabolic disorders characterized by elevated levels of plasma triglycerides. In human patients, HTG is further differentiated depending on the plasma triglyceride levels: mild-to-moderate for triglyceride levels ranging from 2 to 10 mmol/l and severe for concentrations > 10 mmol/l^1^. The main health concern in patients with mild-to-moderate HTG is an increased risk for cardiovascular diseases. Severe HTG additionally poses the risk of developing episodes of acute pancreatitis^1,2^. Severe HTG is often inherited and variants in five different genes have been identified in human patients. The known candidate genes are *APOA5* encoding apolipoprotein A5 (OMIM *606368)^3^, *APOC2* encoding apolipoprotein C2 (OMIM *608083)^4^, *GPIHBP1* encoding glycosylphosphatidylinositol anchored high density lipoprotein binding protein 1 (OMIM *612757)^5^, *LPL* encoding lipoprotein lipase (OMIM *609708)^6^, and *LMF1* encoding lipase maturation factor 1 (OMIM *611761)^7^. Several pathogenic variants in each of these five genes have been reported in human patients with severe HTG.

In domestic animals, to the best of our knowledge, so far only one homologous disease has been characterized at the molecular level. In a cat colony segregating a recessive form of HTG, a missense variant in the *LPL* gene was reported to result in lipoprotein lipase deficiency and chylomicronemia (OMIA 001210–9685)^8^.

Franches-Montagnes (FM) horses are a native breed from Switzerland established at the end of the 19th century with a well-documented breed history^9^. The studbook was officially released in 1921. While originally mainly used as a working horse, introgressions with Arabian and Warmblood horses were carried out in the 1970s and 1980s to develop the FM into a lighter horse type that is well suited for leisure riding and carriage driving. One of these introgressions utilized the Swedish Warmblood stallion Aladin, born in 1964. Alsacien, a son of Aladin and the FM mare Javotte was born in 1969 and became the founder of the so-called L-line, one of the 16 recognized sire lines in modern FM breeding. The FM studbook was closed in 1997^9^. Closed breeding populations necessitate a certain degree of inbreeding, which in turn leads to an increased risk of recessive diseases. In 2024, a mean pedigree-based inbreeding coefficient (F_Ped_) of 6.84% and a mean genomic marker derived inbreeding coefficient (F_ROH_) of 12.15% were reported^9^.

The risk for recessive diseases in closed populations further increases, if popular sires are extensively used in breeding. A well documented example in the FM breed is congenital liver fibrosis (CLF), an autosomal recessive lethal disease with several dozens of reported cases between ~ 1984 and ~ 2013^10,11^. Many of the CLF affected foals were inbred to the stallion Elu, born in 1964 and founder of the E-line^10^. CLF was reported to be associated with the *PKHD1* gene^11^. Although the causal variant for CLF has not been identified, the introduction of an indirect genetic marker test led to a rapid decrease of the CLF prevalence. No further CLF affected foals have been reported since 2013.

The present study was prompted by observations of several FM foals with severely elevated levels of plasma triglycerides that died or were euthanized due to severe acute pancreatitis. We tentatively termed the disease hypertriglyceridemia-induced pancreatitis (HIP). The goal of this study was to investigate a possible genetic cause and to obtain data on the origin and frequency of a potential disease allele.

## Materials and methods

### Ethics statement

All examinations and animal experiments were carried out after obtaining written informed owner’s consent and in accordance with local laws, regulations, and ethical guidelines. Swiss legislation requires that known carriers for hereditary diseases must be identified in the herdbook and that this information must be made available to breeders (Animal Breeding Ordinance, Art. 7)^12^. Blood samples from healthy horses were collected with the approval of the Cantonal Committee for Animal Experiments (Canton of Bern, Switzerland; permit BE94/2022). The study is reported in accordance with the ARRIVE guidelines.

### Animals

This study was conducted with a total of 2122 FM horses born between 1974 and 2024 including 11 HIP-affected horses that are referred to as cases 1 to 11. The phenotype of HIP-affected animals was ascertained by clinical examination, measurement of serum triglyceride levels or postmortem pathological investigation. A plasma triglyceride level above 10 mmol/l was considered sufficient evidence for classification as HIP-affected. If no plasma triglyceride measurements were available, the classification was based on the available clinical and pathological data. Details on the available information for each affected horse are given in Table S1. Biological samples were retrieved from the Vetsuisse Biobank. They represented either leftover diagnostic materials from the Swiss Institute of Equine Medicine (ISME) or the Institute of Animal Pathology (ITPA) or archived material from genetic testing at the Institute of Genetics of the University of Bern. Additional samples from parents of HIP-affected animals were specifically collected for this study.

### Clinical investigations

Nine out of the 11 cases were examined at the ISME equine hospital, two cases had been seen by private practitioners who kindly shared their records with us. Clinical examinations included a physical examination, measurement of blood parameters, analysis of abdominal fluid, and ultrasound examinations. Examinations took place over a time span of 14 years and were not performed in the same manner for all animals. Details are listed in Table S1.

### Euthanasia, necropsy and post mortem examinations

Depending on the individual age, sedation was achieved using either butorphanol at a dose of 0.05 mg/kg (Butomidor, Streuli, Uznach, Switzerland) alone, or in combination with an alpha-2 agonist (0.3 mg/kg Xylasol, Graeub, Bern, Switzerland). After sedation, the foals were placed under short anaesthesia using 2.5 mg/kg ketamine (Ketasol, Graeub, Bern, Switzerland) and diazepam 0.05 mg/kg (Valium, Roche, Basel, Switzerland) before euthanasia with pentobarbital 90 mg/kg (Esconarkon, Streuli, Uznach, Switzerland) was performed. All medication was administered by intravenous Mila Guidewire IV catheter 14 G, except for the only adult individual where a Milacath-extended use catheter 14 G was used (Mila International, Hebron, KY, USA). Post-mortem examinations were carried out in nine out of 11 cases, including a histopathological examination of the pancreas in eight cases (Table S1).

### DNA extraction

Genomic DNA was extracted from EDTA blood using the Maxwell RSC Whole Blood DNA kit in combination with the Maxwell RSC instrument (Promega, Dübendorf, Switzerland). The same instrument was used for DNA isolation from formalin-fixed paraffin-embedded (FFPE) tissue samples with the Maxwell RSC DNA FFPE kit.

### Whole-genome resequencing

An Illumina TruSeq PCR-free library with ~ 410 bp insert size was prepared from case 10. We collected 182 million 2 × 150 bp read pairs on a NovaSeq 6000 instrument (18.9x coverage). Mapping to the EquCab3.0 reference genome assembly was performed as previously described^13^. The sequence data were deposited under study accession PRJEB14779 and sample accession SAMEA115753422 at the European Nucleotide Archive.

### Variant calling and filtering, in silico functional predictions

Variant calling was performed using the GATK HaplotypeCaller^14^ in gVCF mode as previously described^13^. To filter for private variants in the affected horse we used publicly available genome sequences from 69 horses from diverse breeds other than FM^15^ (Table S2). Predictions of functional effects of the called variants were obtained with the SnpEff software^16^ together with the EquCab3.0 reference genome assembly and NCBI annotation release 103. Variants with a SnpEff predicted impact of high or moderate were considered protein-changing variants.

### PCR and Sanger sequencing

The candidate variant, *LMF1*:XM_023616679.1:c.369_373delinsTCT, was genotyped by direct Sanger sequencing of PCR amplicons. A 304 bp (or 302 bp for the mutant allele) PCR product was amplified using AmpliTaqGold360MasterMix (Thermo Fisher Scientific, Waltham, MA, USA) with the addition of 10% 360 GC Enhancer (Thermo Fisher Scientific) to the reaction volume, and primers ECA_LMF1_F and ECA_LMF1_R1 (Table S3). After treatment with exonuclease I and alkaline phosphatase, the sequencing reactions were performed using the reverse PCR primer ECA_LMF1_R1 and an ABI BigDye v3.1 sequencing kit (Thermo Fisher Scientific). Ethanol precipitation was used to purify the sequencing reactions, and an ABI 3730 DNA Analyzer (Thermo Fisher Scientific) was used for analysis of Sanger sequences. The raw sequence data were analyzed with the Sequencher 5.1 software (GeneCodes, Ann Arbor, MI, USA).

### Genotyping by fragment size analysis

The *LMF1* variant was additionally genotyped by fragment size analysis. A fluorescently labelled 128 bp (or 126 bp for the mutant allele) PCR product was generated using QIAGEN Multiplex PCR Master Mix (QIAGEN, Hilden, Germany) with the addition of 20% Q-Solution (QIAGEN) to the reaction volume, and the primers ECA_LMF1_F_FAM and ECA_LMF1_R2 (Table S3). Each PCR product was diluted with 50 µl of RNase-free water. 1 µl of the diluted PCR product was added to 9 µl of HiDi™ formamide (Applied Biosystems by Thermo Fisher Scientific) with 2% GeneScan™ 500 LIZ™ dye size standard (Applied Biosystems by Thermo Fisher Scientific), and the analysis was performed on an ABI3730 capillary sequencer (Thermo Fisher Scientific). Data were analyzed using the GeneMapper 4.0 software (Thermo Fisher Scientific).

## Results

### Phenotypic characterization

**Fig. 1:**
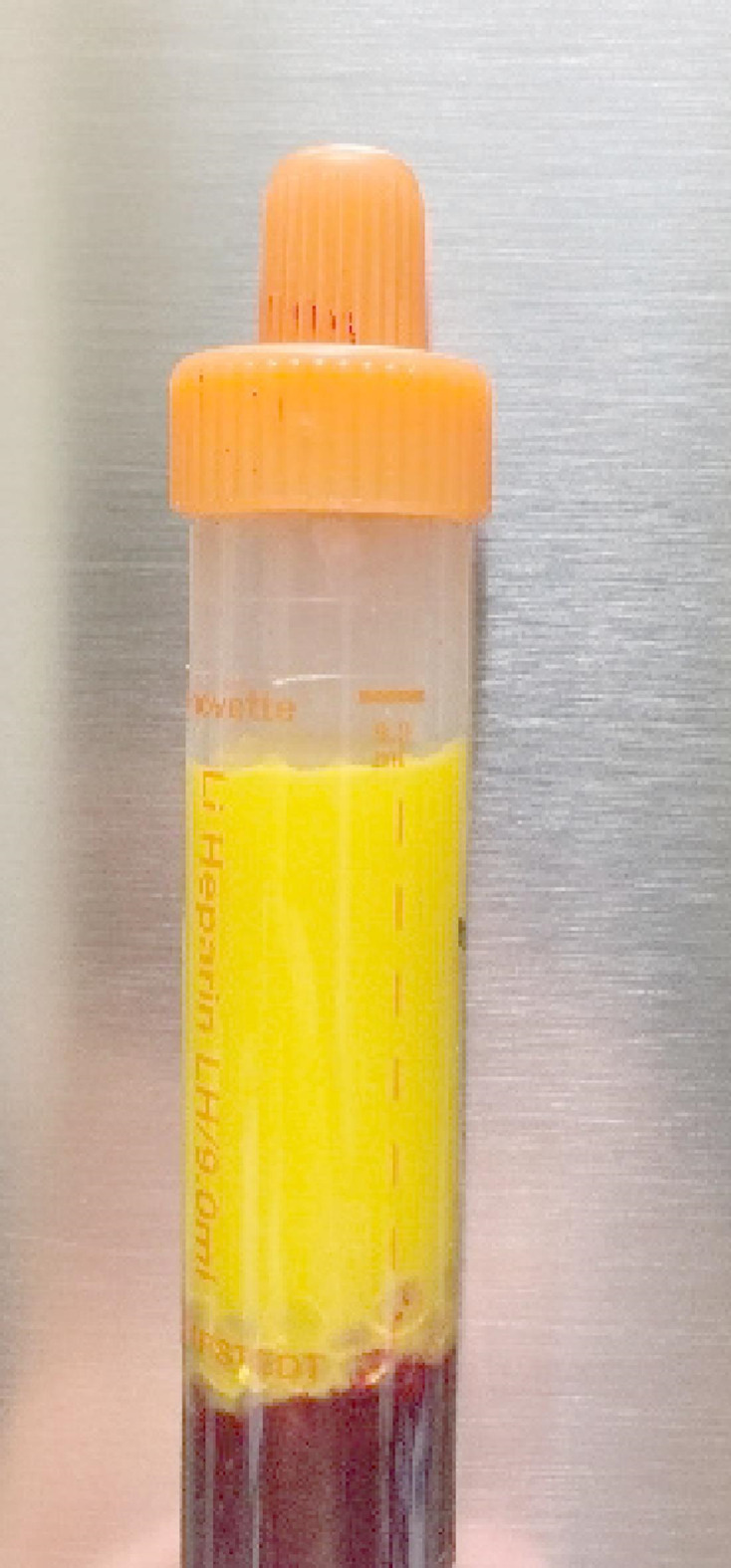
Blood sample of an HIP-affected horse. A large amount of fat resulting from the hypertriglyceridemia is clearly visible.

A total of 11 HIP-affected FM horses were retrospectively identified for this study. In ten of them, plasma triglyceride levels had been measured and found to be severely elevated with values above 10 mmol/l compared to the reference range (0.6–1 mmol/l for foals; 0.17–0.59 mmol/l for adult horses^17^; Fig. 1, Table S1).

Ten of the 11 affected horses died or were euthanized between 6 days and 3 months of age due to severe pancreatitis. The most common clinical signs in affected foals included apathy, reluctance to nurse, abdominal distension and diarrhea, with fever observed in some cases. Other common clinicopathological abnormalities in foals included hypoproteinemia, azotemia, hyperbilirubinemia, hyperlipasemia, and elevated serum amyloid A concentrations. Some foals also had increased liver enzymes.

One affected horse, a 13 year-old gelding, survived until adulthood and presented with a history of chronic weight loss, diarrhea, and intermittent fever, which had acutely worsened. Upon admission to the hospital, the horse showed anorexia, apathy and a high fever of 41 °C. A diagnosis of hepatopathy was made, supported by elevated liver enzymes. The horse was humanely euthanized after several days of treatment due to its lengthy medical history and compromised quality of life.

Pathological examinations in eight affected foals revealed severe, acute to chronic necrotizing pancreatitis and extensive fibronecrotizing peritonitis. The affected adult horse was diagnosed with severe, multifocal, chronic active cholangiohepatitis and acute necrotizing hepatitis, mild chronic pancreatic fibrosis, and mild multifocal chronic peritoneal fibrosis. A more detailed characterization of the clinical and pathological findings is currently in preparation and will be published elsewhere.

### Identification of a candidate causal variant

The pedigrees of the affected horses were highly suggestive for a monogenic autosomal recessive mode of inheritance (see Sect. 3.4 below). We therefore sequenced the genome of an affected foal and searched for homozygous private protein changing variants by comparing the variants to 69 control genomes (Table 1, Table S4). Further prioritization of resulting variants was then done based on known candidate genes for HTG in humans^1,2^.

**Table 1:** Results of variant filtering in an affected horse against 69 control genomes.

Filtering step	Homozygous variants
All variants in the affected horse	2,036,238
Private variants	6,750
Protein-changing private variants	43
in HTG candidate genes (*APOA5*,* APOC2*,* GPIHBP1*,* LPL*,* LMF1*)	1

The bioinformatic analysis identified 43 homozygous private protein-changing variants in the affected horse. Only one of them was located in a functional candidate gene for HTG, in *LMF1*. This variant, Chr13:NC_009156.3:g.42,935,259_42,935,263delinsTCT or XM_023616679.1:c.369_373delinsTCT, leads to an early frameshift in the reading frame of the *LMF1* coding sequence, XP_023472447.1:p.(Leu125Argfs*193). The wildtype equine LMF1 protein contains 561 amino acids and five transmembrane domains. The frameshift is predicted to alter or truncate 436 (78%) of the 561 wildtype codons including the coding information for four of the five transmembrane domains.

### Population screening and genotype-phenotype association

**Fig. 2:**
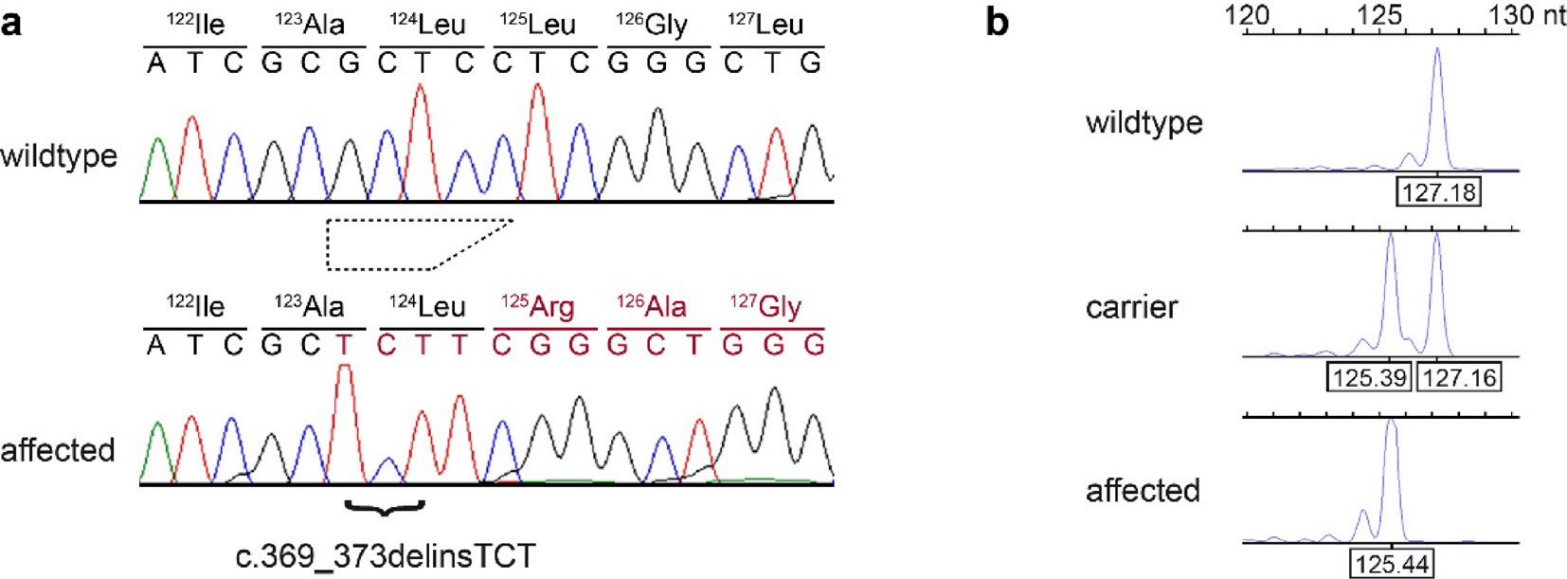
Details of the *LMF1*:c.369_373delinsTCT frameshift variant. (**a**) Sanger electropherograms illustrate the variant that results in a net deletion of 2 nucleotides. The trapezoid indicates that the five wildtype nucleotides GCTCC are replaced by the three nucleotides TCT in the mutant sequence. The depicted wildtype sequence corresponds to Chr13:42,935,254 − 42,935,271 in the EquCab3.0 assembly or Chr13:53,084,083 − 53,084,100 in the TB-T2T assembly. (**b**) Fragment length analysis of fluorescently labeled PCR amplicons was used to genotype large sample numbers for population screening. The indicated sizes were automatically assigned by the GeneMapper software. The true sizes of the wildtype and mutant amplicons are 128 and 126 bp respectively.

The *LMF1*:c.369_373delinsTCT variant was confirmed by Sanger sequencing of PCR amplicons. As the mutant allele is two nucleotides shorter than the wildtype allele, we established a medium throughput genotyping assay based on fragment length analysis of fluorescently labeled PCR amplicons (Fig. 2).

We genotyped a cohort of 2122 FM horses at the *LMF1* variant. At the time of genotyping, this cohort contained 8 HIP-affected horses that had been identified by prior clinical and/or pathological investigations in combination with plasma triglyceride measurements. They all carried the mutant *LMF1* allele in a homozygous state. The cohort contained 3 additional horses with a homozygous mutant *LMF1* genotype that had not been recognized as HIP-affected prior to the genetic investigation. Two of the homozygous mutant animals were foals that had previously been submitted for CLF testing. They both tested negative for CLF and died at 23 days or 6 weeks of age, respectively. Retrospectively, the available clinical and pathological data were compatible with HIP (Table S1). The remaining homozygous mutant *LMF1* mutant horse was the aforementioned 13-year old gelding. Upon clinical examination, this horse had severe HTG and was in a poor health condition that led to euthanasia. We therefore concluded that all 11 homozygous *LMF1* mutant horses were affected by HIP. None of the other 2111 tested horses carried a homozygous mutant *LMF1* genotype.

Taken together, we found a perfect association of the *LMF1* genotypes with the HIP phenotype in the cohort of 2122 FM horses (p_Fisher_ = 1.0 × 10^–29^; Table 2). The mutant allele had a frequency of 7.51% in the control cohort, which corresponds to a carrier frequency of 15.0%. The observed genotype frequencies were in Hardy-Weinberg equilibrium (p_HWE_ = 0.45).

**Table 2:** Genotype-phenotype association at the *LMF1*:c.369_373delinsTCT variant.

Phenotype	wt/wt	wt/mut	mut/mut
HIP-affected horses (*n* = 11)^a^	-	-	11
Controls (*n* = 2111)	1794	317	-

^a^Phenotype classification was based on plasma triglyceride levels > 10 mmol/l for 10 of the 11 HIP cases. In one animal (case 5), plasma triglyceride levels were not measured, but the foal had died at 6 weeks of age and the clinical history was consistent with HIP (details are given in Table S1)

.

### Pedigree analyses and origin of the mutant allele

**Fig. 3:**
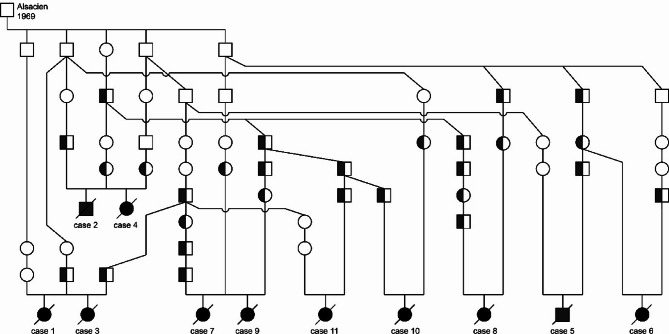
Pedigree of HIP-affected foals indicate inbreeding to a common ancestor. A total of 11 HIP-affected FM horses were ascertained (filled symbols). All 11 horses carried the mutant *LMF1* genotype in a homozygous state and were inbred to the stallion Alsacien, born in 1969, an important stallion and founder of the so-called L-line within the FM breed. Half-filled symbols indicate animals whose heterozygous genotype and carrier status was confirmed by DNA testing. Unfilled symbols indicate unaffected horses, from which no DNA for genotyping was available. Please note that this pedigree shows only a subset of ancestors of affected horses representing the most likely connecting paternal and maternal lineages to Alsacien.

All 11 HIP cases descended from unaffected parents. They comprised three pairs of maternal or paternal half-siblings, which had initially raised the first suspicion of a hereditary disease. A deeper analysis of the pedigrees revealed that all HIP cases were inbred to the stallion Alsacien within 4–9 generations. The pedigree data were thus fully compatible with a monogenic autosomal recessive mode of inheritance. DNA samples from 28 hypothetical carriers based on their pedigree were available for testing and for each of them the heterozygous *LMF1* genotype was experimentally confirmed. Thus, the pedigree showed the expected genotype-phenotype co-segregation (Fig. 3).

## Discussion

In this study, we identified an *LMF1* frameshift variant in 11 FM horses with HIP. The encoded lipase maturation factor 1 is a transmembrane protein in the endoplasmic reticulum required for the correct posttranslational maturation of the enzymes lipoprotein lipase (LPL) and hepatic lipase (HPL)^2,18,19^. The phenotype resulting from loss-of-function variants in *LMF1* has been termed combined lipase deficiency in human patients and the *cld* mouse mutant^7,20^.

LPL mediates intravascular hydrolysis of triglycerides packed in lipoproteins such as chylomicrons and very-low-density lipoprotein (VLDL) into fatty acids^18^. As LMF1 is essential for the secretion of enzymatically active LPL, deficiency of functional LMF1 leads to severely elevated plasma triglyceride levels in human patients, frequently resulting in episodes of acute pancreatitis^2,21–23^. Although the exact pathogenesis of hypertriglyceridemia-induced pancreatitis in humans remains incompletely understood, two complementary mechanisms have been discussed. One suggests that the accumulation of lipoproteins in the bloodstream increases blood viscosity and impairs blood flow in the pancreatic capillaries, which may lead to hypoxic conditions and release of pancreatic lipase into capillaries. The second proposed mechanism involves the hydrolysis of triglycerides by pancreatic lipase and release of free fatty acids, which subsequently trigger an inflammatory cascade^24^. In human patients, the disease can be managed by strictly controlling the dietary intake of triglycerides^1,24^. However, only very few human patients with characterized *LMF1* variants have been reported in the literature, and any possible variability of the human clinical phenotype has probably not yet been fully characterized^7,21–23^.

In neonatal Lmf1-deficient *cld* mice, plasma triglycerides steeply increase after the ingestion of dietary milk. The severe HTG causes an increase in blood viscosity, ischemia and cyanosis. The inability of tissues to access circulating triglycerides results in starvation, poor thermoregulation and death 2–3 days after birth^7^.

The HIP phenotype in FM horses shows many parallels to the phenotypes in LMF1-deficient human patients and *cld* mice. Hallmark of the phenotype is the severe HTG with plasma triglyceride levels > 10 mmol/l^1^. The severe HTG may lead to episodes of acute necrotizing pancreatitis, resulting in death at a very young age in most affected horses.

We acknowledge limitations of our study. The genetic analyses were conducted in parallel to ongoing clinical investigations. Three of the eleven presumed HIP cases were identified by genotyping and only retrospectively phenotyped. While the available phenotypic information seemed compatible with the HIP phenotype in all 11 cases, HTG was not confirmed in case 5. The only available phenotypic information on case 5 comprises death at 6 weeks of age with pathological changes of the liver. The pancreas of this foal was not examined. The liver of this foal was submitted to pathological investigation as the attending veterinarian suspected CLF, which was ruled out by the pathological investigation of the liver and genotyping. As the age of death and the available clinical history of case 5 matched the findings of the other HIP cases, we classified it as HIP affected despite lacking a plasma triglyceride measurement.

According to the ACMG/AMP consensus criteria for human diagnostics^25^we compiled the following arguments for the pathogenicity of the *LMF1* variant: it is a frameshift variant that may be assumed to result in a null allele (PVS1), the prevalence of the mutant allele is significantly increased compared to controls (PS4), and perfect genotype-phenotype co-segregation was observed in a very large pedigree (PP1). These arguments are sufficient to designate the *LMF1*:c.369_373delinsTCT variant as pathogenic. We think that the presented genetic data and confirmation of the metabolic defect with elevated triglyceride levels in affected horses combined with the existing knowledge on *LMF1* and the effects of loss-of-function variants in other species^7,21–23^ proves the causality of the variant for the HIP phenotype.

Due to the availability of excellent pedigree records and many DNA samples, we were able to trace the origin of the mutant allele with relatively high confidence. However, it must be cautioned that the hypothetical carrier status of Alsacien could not be confirmed by genotyping as no DNA sample from this stallion was available. Furthermore, any errors in the pedigree records might also have confounded our analysis. Alsacien was an important stallion in the recent development of the FM breed, especially through his sons that were influential since the 1970s. As an F1 Warmblood x FM stallion, he was mainly responsible for developing the former draft horse breed into a lighter horse type suitable for leisure activities. Assuming that Alsacien was indeed a carrier, it is not surprising that the mutant allele has reached a frequency of more than 7% in the current FM population.

The clinical phenotype of HIP affected horses is quite unspecific if no bloodwork is performed. Young foals may die with similar clinical signs due to many causes. CLF in FM horses is characterized by a progressive liver failure that results in a similar clinical phenotype, which also has a variable age of onset^10,11^. It is thus conceivable that in the past HIP affected foals may have been erroneously assumed to be affected by CLF, which then in turn may have delayed the recognition of HIP as a new hereditary disease in the breed. Given the Warmblood ancestry of Alsacien, it cannot be excluded that the HIP allele originated in Warmblood horses. Further research is warranted to make sure that it does not or no longer segregate in Warmblood horses as well.

CLF and HIP do not only share a related clinical phenotype. The population dynamics and history of these recessive diseases is remarkably similar and involved the intense use of two popular stallions, which may have led to the spreading of recessive disease alleles. The stallion Elu, born in 1964 and founder of the E-line, likely contributed to the massive increase of the CLF allele frequency in the FM population that resulted in a wave of CLF cases between ~ 1984 and ~ 2013^10,11^. Alsacien, the founder of the L-line was born in 1969 and is likely responsible for the spread of the HIP allele, which gave rise to a series of HIP cases that has been going on for at least the last 15 years. These findings emphasize the importance of good breeding practices, surveillance of newly arising hereditary diseases and monitoring of the inbreeding situation in closed domestic animal populations.

In conclusion, this study identifies HIP as a new autosomal recessive inherited disease in the FM horse breed. We identified the *LMF1*:c.369_373delinsTCT frameshift variant as causal variant. Our findings provide a spontaneous large animal model for the homologous human disease that may shed new insights into the physiology of lipid metabolism. The identification of the causal variant enables genetic testing and avoiding carrier x carrier matings to prevent the unintentional breeding of further HIP-affected foals.

## Supplementary Information

Below is the link to the electronic supplementary material.

Supplementary Material 1

Supplementary Material 2

Supplementary Material 3

Supplementary Material 4

Supplementary Material 5

## Data Availability

All data are contained in this manuscript and the supplementary files. Accession numbers for the whole genome sequence data are given in Table S2.
