# Supplementary material for: LMF1 frameshift deletion in Franches-Montagnes horses with hypertriglyceridemia-induced pancreatitis
Source: Sci Rep. 2025 Aug 6;15:28667. doi: 10.1038/s41598-025-13954-9 (PMC12326015; doi:10.1038/s41598-025-13954-9)
Supplement: Supplementary file 5 — Supplementary Material 5 [file 41598_2025_13954_MOESM5_ESM.docx]

Supplementary information

Table S1. Information on 11 HIP-affected horses in this study.

Table S2. Accession numbers of whole genome sequences of 70 horses.

Table S3. Primer sequences.

Table S4. Private variants in the genome of the sequenced case.
